# Relief of the negative effects of heat stress on semen quality, reproductive efficiency and oxidative capacity of rabbit bucks using different natural antioxidants

**DOI:** 10.5713/ajas.20.0258

**Published:** 2020-06-24

**Authors:** Ibrahim Talat El-Ratel, Kandil Abdel Hai Attia, Ali Ali El-Raghi, Sara Fikry Fouda

**Affiliations:** 1 Department of Poultry Production, Faculty of Agriculture, Damietta University, 34517, Damietta, Egypt; 2 Department of Evaluation of Natural Resources, Environmental Studies and Research Institute, El-Sadat City University, Cairo, 32897, Egypt; 3 Department of Animal Production, Faculty of Agriculture, Damietta University, 34517, Damietta, Egypt; 4 Department of Poultry Production, Faculty of Agriculture, Mansoura University, 35516, Mansoura, Egypt

**Keywords:** Rabbit Bucks, Heat Stress, Natural Antioxidant, Semen Quality, Seminal Plasma Constituents

## Abstract

**Objective:**

The potential of extra virgin olive oil (EVOO), betaine (BET), and ginger (GIN), as natural antioxidants, in reducing negative effects of heat stress on physiological responses, antioxidant capacity, semen quality and fertility of bucks under heat stress were investigated.

**Methods:**

Forty adult Animal Production Research Institute line rabbit bucks were distributed randomly into four experimental treatments of ten rabbits each. The first treatment was fed the commercial pellet diet (CPD) without supplementation and served as a control. The other three treatments were fed CPD supplemented with EVOO (300 mg), BET (1,000 mg), and GIN (200 mg) per kg diet for 3 consecutive months during the summer season.

**Results:**

Supplementation of EVOO, BET, or GIN improved (p<0.05) the sexual desire, progressive motility, vitality, intact acrosome and membrane integrity, sperm cell concentration, sperm outputs and fertility. Seminal plasma total proteins, globulin, total antioxidant capacity, glutathione and glutathione S-transferase, and initial fructose increased (p<0.05), while total lipids, aspartate and alanine aminotransferases and malondialdehyde decreased (p<0.05) compared with the control. In comparing the natural antioxidants treatments, GIN evoked the largest improvement.

**Conclusion:**

The inclusion of GIN (200 mg/kg diet) appeared to improve the sexual desire, semen quality and oxidative stress of bucks. This may be a beneficial supplement for the management of rabbit bucks used in natural mating or artificial insemination.

## INTRODUCTION

In comparing with other livestock, rabbit are characterized small body size, efficient feed utilization, rapid growth rate, high quality nutritious meat, early maturity, high reproduction rate and high genetic selection potential [1]. The environmental and nutritional factors are affecting the economic intensive rabbit production. Under environmental temperature more than 25°C, reduce their productivity by several behaviour and physiological changes [2]. The reproductive efficiency of bucks is economically important because semen with high characteristics is required for avoiding the loss in valuable genotypes and achieving high fertility [3]. Oxidative stress plays an important role in sperm motility, function, quality and fertility, since lipid peroxidation increases under heat stress (HS) [4]. Improper environmental conditions lead to reduction in quality and fertility of sperm cells, so HS negatively influences the testicular function [5].

Rabbit spermatozoa display high metabolic activity and they are rich in polyunsaturated fatty acids in plasma membrane, which may cause increasing lipid peroxidation [6], so they are sensitive to free radicals attacks. This may result in reducing sperm motility, fragmentation of DNA and reducing sperm fertilizing ability [4]. Also, an excessive free radicals production exceeds the anti-oxidant capacity of the seminal plasma, leading to damaged mitochondria and membranes (acrosomal and plasma) of spermatozoa [7]. Therefore, several strategies, like management, nutrition and hormones have been adopted to eliminate the harmful effects of HS [8]. In this respect, there is a recent attention towards the usage of natural antioxidants to counter negative impacts of HS on semen quality of males by limiting free radical production, with ease at an affordable price and without side effects [4,6].

Ginger (GIN, *Zingiber officinale*, family Zingiberaceae) is common medicinal plant with several health benefits around the world, and its extract contain different active phytochemical components such as volatile oils, gingerol, gingerone, piperine, shogaols, and zingerone [9]. It also contains polyphenols (flavonoids and flavones glycosides) and anti-oxidants like B-carotene, ascorbic acid, terpenoids and alkaloids, thus it has androgenic and anti-oxidative properties [10]. The GIN has been used to improve the reproductive efficiency and antioxidants status of rabbit bucks [11] under normal conditions.

Olive (Olea europaea, Oleaseae family), is a very popular plant for its biological and pharmacological traits, and its oil contains mono-unsaturated fatty acids (77%), saturated fatty acids (14%), polyunsaturated fatty acids (9%), alpha-tocopherol and vegetable mucilage [12]. It has beneficial bioactive components such as flavonoids (luteolin and apigenin), polyphenols (oleuropein, hydroxytyrosol, and tyrosol) and squalene, as bioactive organic molecules. These compounds have antimicrobial, antioxidant and anti-inflammatory properties [13].

Betaine (BET) is a trimethyl-glycine produced by choline oxidation and it is implicated in methionine and choline sparing, in fat distribution and immune responses [14]. The BET has been found to alleviate HS in roosters [4] and in growing rabbits [15]. Under HS, BET has been found to improve immunity and health status in laying hens [16], but studies on the reproduction of rabbit bucks remain limited. According to the available literature, studies on impact of different natural antioxidants with various modes of action to relieve the negative effects of HS on rabbit bucks are scarce. As such, limited studies addressed the comparison of the effect of among extra virgin olive oil (EVOO), BET, and GIN on reproductive strategy of rabbit bucks under HS.

Therefore, the objectives of this study were to investigate the efficacy of dietary supplementation with different types of natural antioxidant materials including EVOO (300 mg), BET (1,000 mg), or GIN (200 mg) per kg diet to improve physiological responses, oxidative capacity, semen quality and fertility of rabbit bucks exposed to HS in summer conditions.

## MATERIALS AND METHODS

The experimental work of this study was conducted at a private commercial rabbit farm, Mansoura City, Dakahlia Governorate, Egypt. The laboratorial work was carried out at Physiology and Biotechnology Laboratory, Animal Production Department, Faculty of Agriculture, Mansoura University, Egypt. The experimental procedures were conducted according to the Directive 2010/63/EU of the European Parliament and of the Council of 22 September 2010 on the Protection of Animals Used for Scientific Purposes.

### Animals

Forty sexually mature Animal Production Research Institute (APRI) line rabbit bucks with 8 month-old and an average live body weight (LBW) of 3.05±0.22 kg were used in this study. Bucks were individually housed in stainless steel cages batteries (40×50×35 cm) accommodated with feeders for pelleted rations and automatic fresh-water drinkers within a naturally ventilated and lighted rabbitry. The experimental bucks were fed *ad libitum* on a commercial pellet diet (CPD) covering the daily nutritional requirements of rabbit bucks according to the national research council guidelines [17]. The ingredients and chemical analysis of CPD is represented in Table 1.

### Experimental design

Bucks were distributed randomly into four homogenous experimental treatments (10 in each), in a straight-run experimental design. Bucks in the first treatment were fed the CPD without supplementation and served as the control. Bucks in the other three experimental treatments were fed the CPD supplemented with 300 mg of EVOO (ILIADA PDO Kalamata EVOO; AGRO. VI. M.S.A., Kalamata, Greece), 1,000 mg of BET (natural betafine, Adisseo, France) and 200 mg of GIN per kg, respectively. The weekly CPD of each treatment was well mixed with their additives in homogenous form. Bucks were fed the experimental diets throughout experimental treatments of 3 consecutive months from 1st June to 30th September.

### Climatic condition

Throughout the experimental treatments, ambient temperature (AT) and relative humidity (RH) were recorded daily at 1 p.m. using a hydro-thermograph located inside the rabbitry. The daily values of AT and RH were estimated. Temperature-humidity index (THI) was calculated according to the following equation [18]:

$$THI=T-\left[\left(0.31-0.31\left(\frac{RH}{100}\right)\right)(T-14.4)\right]$$

Where T, dry bulb temperature (°C) and RH, relative humidity. THI values of <27.8 (absence of HS); 27.8 to 28.9 (moderate HS); 29.0 to 30.0 (severe HS); and >30.0 (very severe HS).

Table 2 show AT, RH, and THI during the experimental treatments. Results of the calculated THI value indicate that the experimental bucks were under HS, being severe during June-August and moderate in September.

### Body weight and feed intake

The LBW (kg/buck) and feed intake (g/buck) were recorded for the entire experimental treatments.

### Physiological response parameters

The temperature of skin (ST), rectal (RT), and ear (ET) were individually measured, at the same time of measuring AT and RH, using a digital thermometer (Type “K” Thermocouple, ±0.01°C). The ST was measured at one location between the loin and neck on the body surface. The RT was measured by inserting the probe of thermometer at a depth of 2 cm into the rectum. The ET was measured by placing the probe of digital thermometer in direct contact with the internal central area of the auricle. The measurement durations were minimized as possible to be all in similar times.

### Semen collection and evaluation

Rabbit bucks were trained for semen collection by artificial vagina prior to the main collection period. From 1st August to 30th September, as semen collection period, semen was collected from all rabbit bucks (n = 10) in each group twice/week for 9 successive weeks. Therefore, 180 ejaculates (10 bucks ×2 ejaculates/h/wk×9 weeks) were collected and evaluated per group, and then the weekly data of each semen parameter were averaged for each rabbit during the whole collection period to avoid using them as experimental replications.

On day of collection, ejaculates were obtained in the morning (8 a.m.) by using an artificial vagina of rabbits (40°C to 41°C) and a teaser doe. The reaction time was recorded by measuring the time elapsed from a doe insertion into the cage of buck until complete ejaculation (as an indication of libido). After collection, the semen volume of each ejaculate was recorded using a graduated collection tube after removal of the gel mass and semen pH value was immediately determined using a pH paper (Universalindikator pH 0 to 14 Merck, Merck KgaA, 64271 Darmstadt, Germany). Semen was maintained in a water bath (37°C) and then transferred to the laboratory for semen evaluation. Special attention was given to protect semen from cold or heat shocks and direct light. Throughout the course of semen collection, time and place of collection, and collector were kept constant.

Semen was diluted with saline (0.9% Nacl) at a rate of 1 semen: 10 saline, then percentage of sperm progressive motility was determined in five microscopic fields per semen sample using a phase-contrast microscope (Leica DM 500, Leica Mikrosysteme Vertrieb GmbH, Wetzlar, Germany) supplied with a hot stage at 37°C. Aliquots of raw semen (5 μL) were fixed using a vital stain of eosin (5%) and nigrosine (10%) to determine percentages of vitality, abnormality and normality in 200 spermatozoa in 5 microscopic fields by using phase contrast microscopy (Leica DM 500, Leica Mikrosysteme Vertrieb GmbH, Germany) at 400× magnification. Vital sperm cells (unstained sperm cells) and morphological abnormality (head or tail defects) were examined. Sperm cell concentration (×10^6^/mL) was determined after semen dilution (1 semen: 99 saline) by using the improved Neubauer hemocytometer slide (GmbH +Co., Brandstwiete 4, 2000 Hamburg 11, Germany). The percentages of sperm cells with intact acrosome were evaluated in dried semen smears stained with naphthol yellow S and erythrosine B. The presence or absence of the acrosomal cap in 200 sperm cells were recorded and classified to intact and non-intact acrosome sperm cells.

The hypo-osmotic swelling test (HOS-t) was used to evaluate the functional integrity of the sperm plasma cell membrane. The assay was performed by incubating a mixture of 30 μL semen with 300 μL hypo-osmotic solution (0.90 g fructose+0.49 g sodium citrate/100 mL of distilled water; osmolarity of 50 mOsm/L) in a water bath (37°C) for 30 minutes. Spermatozoa with swollen and curled tails (membrane integrity), were considered responded to HOS-t and were calculated in 200 sperm cells by using phase contrast microscopy (Leica DM 500, Leica Mikrosysteme Vertrieb GmbH, Germany) at 400× magnification. Sperm outputs as total (TSO), motile (MSO), normal (NSO), vital (VSO) and functional (FSO) calculated according to the following equations: TSO = net semen volume (mL) ×sperm cell concentration (×10^6^/mL); MSO = progressive motility %×TSO.

$$\begin{array}{l} \text{NSO}=\text{normality}\%\times \text{TSO};\\ \text{VSO}=\text{vitality}\%\times \text{TSO};\\ \text{FSO}=\text{MSO}\times \text{NSO}\times \text{VSO}\times \text{TSO.} \end{array}$$

### Fertility study

Total of 120 sexually mature APRI rabbit does were distributed into four groups. Does in each group (n = 30) were naturally mated with five bucks from each treatment (6 does/buck) at 3 days mating interval. Reproductive criteria including pregnancy and parturition rates and litter size at birth (total born and total born alive) and at weaning (on day 28 of age) were recorded, then viability rate of kits at birth and weaning was calculated. Pregnancy diagnosis was performed manually by abdominal palpation to calculate pregnancy rate (PR) using the following equation: PR = (number of pregnant does/number of mated does) ×100. After birth, parturition rate (PR) was calculated as the following equation: PR = (number of delivered does/number of pregnant does) ×100.

### Analytical procedures

At the last week of semen collection, seminal plasma were separated by centrifugation at 1,500 rpm for 20 min and stored at −20°C, pending biochemical analysis. After collection, concentration of initial fructose, total proteins (TP), albumin (AL), and total lipids were determined. However, globulin (GL) was calculated by subtracting the AL values from the corresponding TP values. Activity of aspartate aminotransferase (AST), alanine aminotransferase (ALT), alkaline phosphatase (ALP), and acid phosphatase (ACP) in seminal plasma were determined. In addition, total antioxidant capacity (TAC), glutathione content (GSH), glutathione peroxidase (GPx), glutathione S-transferase (GST), superoxide dismutase (SOD), and malondialdehyde (MDA) were assayed. Concentration of biochemicals, enzyme activity and oxidative capacity were determined by using commercial available kits (Bio-diagnostic Co., Recycling Crusher-SBM, www.Bio-diagnostic.com) and spectrophotometer (Spectro UV-VIS Auto, UV-2602, Labomed, Los Angeles, CA, USA).

Blood samples were collected from five bucks in each group from ear vein into heparinized tubes and were placed immediately on ice box. Plasma was obtained by blood centrifugation at 3,000 rpm for 20 min and stored at −20°C until assaying the testosterone concentration by enzyme-immunoassay using commercial kits (Biosource-Europe S.A. 8, rue de L’Lndustrie.B-1400 Nivelles, Belgium). The intra- and inter-assay coefficients of variation were 7.8% and 8.4%, respectively. The minimum detectable limit was 0.1 ng/mL and the maximum limit was 18.0 ng/mL.

### Statistical model and analysis procedure

Data were subjected to analysis of variance using general linear model procedure (GLM) of statistical analysis system SAS [19] (Cary, NC, USA).

The following statistical model was applied for analysis of all measurements

$${\text{Y}}_{\text{ij}}=\mu +{\text{TRT}}_{\text{i}}+{\text{e}}_{\text{ij}}.$$

Where, Y_ij_ = Observations, μ = Overall mean, TRT = effect of ith antioxidant material (i, 1 to 4), e_ij_ = random error. The differences between the control and antioxidants were investigated according to orthogonal comparisons, while odds ratio and 95% confidence intervals for pregnancy and parturition cases were determined according to binary logistic regression using the same program mentioned before. The differences between treatment means were separated by Tukey’s studentized range (HSD) test. The statistical significance was accepted at p<0.05. Shapiro-Wilk test was conducted in order to check for normality [20]. The association between natural antioxidants supplementation and each of pregnancy rate and parturition rate was detected by Chi-Square test (χ^2^). Person correlation coefficients were done according to CORR Procedure within SAS program [19].

## RESULTS

### Performance and physiological response

Based on the results presented in Table 3, the effect of treatment with different types of natural antioxidants (EVOO, BET, and GIN) was not significant (p>0.05) on the mean final LBW and feed intake of rabbit bucks during the experimental period. In comparing with other treatments and the control, only BET supplementation significantly (p<0.05) reduced rectal, skin and ear temperature degrees.

### Libido of rabbit bucks

**Figure 1:**
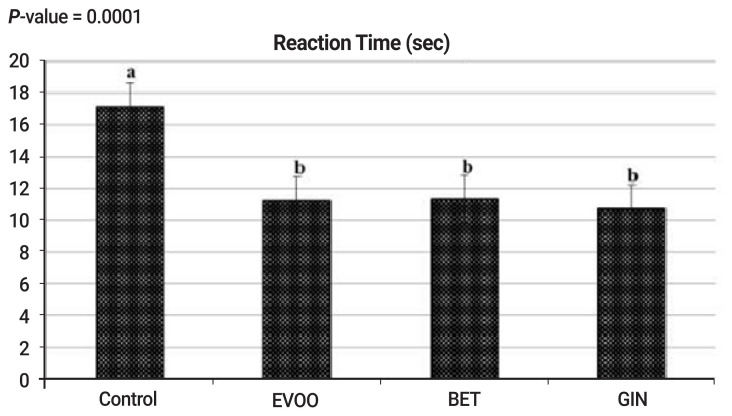
Effect of extra virgin olive oil (EVOO), betaine (BET), and ginger (GIN) supplementation on reaction time of rabbit bucks under heat stress (n = 10 in each group). * p-value of treatment was 0.0001 according to one-way analysis of variance.

**Figure 2:**
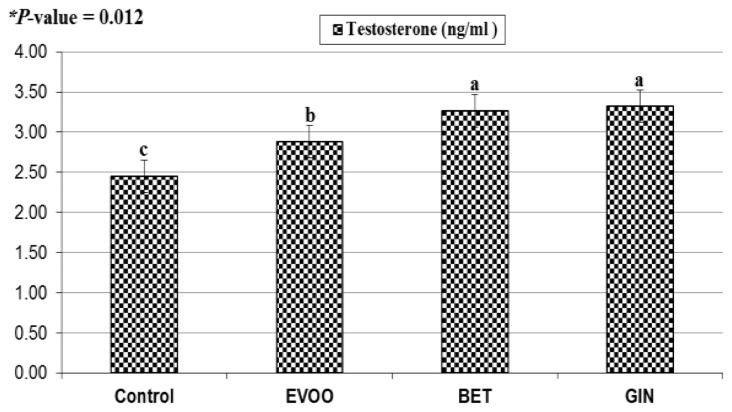
Effect of extra virgin olive oil (EVOO), betaine (BET), and ginger (GIN) supplementation on plasma testosterone concentration of rabbit bucks under heat stress (n = 5 in each group. * p-value of treatment was 0.012 according to one-way analysis of variance.

Results of Figures 1 and 2 show that the effect of natural antioxidants (EVOO, BET, and GIN) supplementation significantly (p<0.05) decreased reaction time (Figure 1) and significantly (p<0.05) increased blood plasma testosterone (Figure 2) compared with the control. Correlation coefficient between testosterone and reaction time was significantly negative (r = −0.89, p<0.0001). This reflects positive impact of EVOO, BET, and GIN supplementation on improving the sexual desire of rabbit bucks, particularly GIN supplementation.

### Semen production

According to the results of Table 4, EVOO, BET, or GIN supplementation significantly (p<0.05) improved semen production, quantitatively and qualitatively. Net semen volume, percentages of progressive motility, vitality, intact acrosome and membrane integrity, sperm cell concentration, TSO, MSO, NSO, VSO, and FSO increased (p<0.05), while percentage of tail abnormality decreased (p<0.05) in treatments compared to the control. On the other hand, semen pH value increased (p<0.05) by GIN and BET, while, head abnormality percentage decreased (p<0.05) only by GIN supplementation as compared to the control. In comparing the natural antioxidants treatments, GIN evoked the largest (p<0.05) improvement in semen production parameters, followed by BET and EVOO, respectively.

### Biochemicals, enzyme activity and oxidative capacity in seminal plasma

The effect of dietary EVOO, BET, or GIN supplementation on biochemical constitutes, enzyme activity, and oxidative capacity in seminal plasma of rabbit bucks is presented in Table 5. The natural antioxidants (EVOO, BET, or GIN) supplementation increased (p<0.05) concentration of TP, GL, and initial fructose compared to the control. However, total lipids showed an opposite trend. Activity of AST and ALT was significantly (p<0.05) decreased, while ALP activity was significantly (p< 0.05) increased by EVOO, BET, or GIN supplementation in comparing with the control. Concentration of AL was significantly (p<0.05) increased only by GIN compared with the control and other treatments. However, AL/GL ratio and ACP activity were not affected significantly (p>0.05) by EVOO, BET, or GIN treatment. In comparing all the natural antioxidants studied, GIN evoked additional reduction (p<0.05) in total lipids, AST and ALT compared with EVOO and BET. Respecting to the effect of the natural antioxidants addition on oxidative capacity (Table 5), it can be observed that the inclusion of EVOO, BET, or GIN significantly (p<0.05) increased levels of GSH and GST, and significantly (p<0.05) decreased MDA level as compared to the control. While, TAC level significantly (p<0.05) increased by BET and GIN supplementation compared with EVOO and the control. However, GPX and SOD were not affected significantly (p> 0.05) by the natural antioxidants additives.

### Fertility

**Figure 3:**
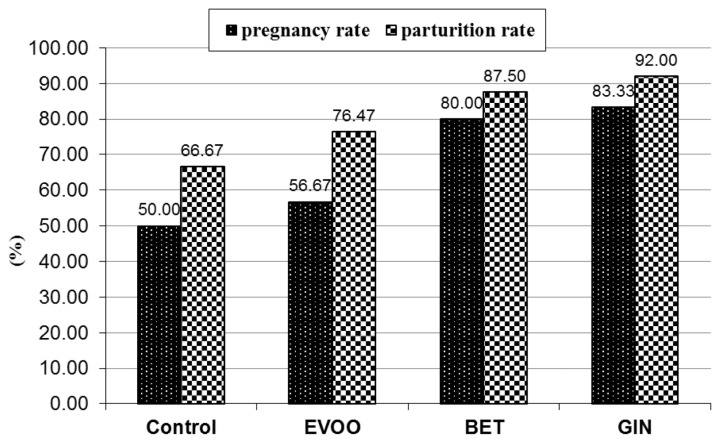
Pregnancy and parturition rates of rabbit does naturally mated by bucks treated with extra virgin olive oil (EVOO), betaine (BET), and ginger (GIN). (See Table 7 for doe numbers of total, mated, and delivered does). * p-value was 0.009 for pregnancy rate and 0.167 for parturition rate according to χ^2^ −test.

Results illustrated in Figure 3 clearly indicated that pregnancy rate of does mated by bucks treated with BET and GIN significantly (p<0.05) increased compared with EVOO and the control. Meanwhile, parturition rate showed non-significant differences, although does mated by bucks treated with GIN showed higher parturition rate than the control, followed by BET and EVOO due to the differences in the numbers of pregnant rabbit does between different treatments. Calculated values for odds ratios indicated that pregnancy cases of does mated by bucks treated with EVOO, BET, and GIN were higher 1.3, 4.0, and 5.0 times than the control, respectively. The corresponding values of parturition cases were higher 1.63, 3.5, and 6.3 times than the control (Table 6).

Total and live litter size at birth as well as litter size at weaning significantly (p<0.05) improved in rabbit does mated by bucks received dietary natural antioxidants compared to the control, but non-significant differences were observed between GIN and BET treatments. Generally, does mated by bucks treated with GIN showed the highest litter size at birth and weaning. Viability rate at birth was significantly (p<0.05) improved only for kits produced by does mated by bucks in GIN treatment, while viability rate at weaning was not affected significantly (p>0.05) by treatment (Table 7).

## DISCUSSION

In tropical and subtropical regions, alleviation of the deleterious effects of HS is the most challenges of animal production. The HS can evoke multiple biological and physiological responses [2]. Under HS conditions, reactive oxygen species were increased, while antioxidant capacity decreased leading to oxidative stress [21]. Oxidative stress negatively affects semen quality and sperm function, as a result of lipid peroxidation in the plasma membrane [4]. The HS negatively influences the testicular function, which may suppress testosterone production [5] lead to reduction in quality and fertility of sperm cells. Damage of sperm DNA and decreased motility, membrane integrity, antioxidant defense system and fertility are the main causes of oxidative stress [10]. In the rabbit production, productivity of animals was affected by HS, which is considered as an important stressor [8]. According to the climatic conditions in the environment of rabbit bucks in our study, the calculated THI value indicates that the experimental bucks were under HS, being severe during June-August and moderate in September. The supplementation of natural antioxidants is essential to relief the adverse effects of HS [15].

In our study, the hypothesis is that HS negatively affects physiological responses, antioxidant capacity, immunity, semen quality and fertility of rabbit bucks, and supplementation of natural antioxidants such as EVOO, BET, or GIN may show promising relieving effects in improving semen quality and sperm fertility of rabbit bucks. Antioxidant supplementation improved the sexual desire of rabbit bucks, which was depressed by HS in the control bucks, through suppress testosterone production, causing destroy Leydig cell function [22]. In this concern, antioxidant supplementation increased testosterone profile and reduced reaction time by improving the testosterone synthesis [23]. In addition, antioxidant administration (EVOO, BET, or GIN) improved semen production, quantitatively and qualitatively, including net semen volume, motility, vitality and normal morphology of spermatozoa, acrosomal and membrane integrities, sperm cell concentration, as well as TSO, MSO, NSO, VSO, and FSO, which adversely affected by HS in rooster [4].

This improvement was mainly related to their properties as natural antioxidants. In this respect, all EVOO, BET, and GIN decreased MDA level as lipid peroxidation marker, while increased TAC in term of GSH and GST contents in the seminal plasma. These antioxidants act as ROS scavengers within the testicular tissues by decreasing the oxidative damage in tissues through the protective effects of antioxidant enzymes, which play an important modulatory role against endogenous oxidative damage [10]. It is worthy noting that natural antioxidants supplementation (EVOO, BET, or GIN) improved the chemical composition of the seminal plasma from TP, AL, GL and initial fructose, while decreased total lipids, AST and ALT, which may has a vital role in function and metabolism of sperm cells. Similar results were reported by El-Speiy et al [10], who found that dietary treatment with natural antioxidant (0.5% or 1% GIN) resulted in higher TP, AL, and GL levels, and lower AST and ALT activity in rabbit seminal plasma. The observed reduction in AST and ALT activities in seminal plasma of treated bucks is in association with improving membrane integrity of spermatozoa [24].

The obtained results indicated the highest antioxidant properties for GIN administration in comparing with EVOO and BET. In this context, GIN extract contain different active phytochemical components such as volatile oils, gingerol, gingerone, piperine, shogaols,acid, terpenoids, and zingerone [9]. It contains polyphenols (flavonoids and flavones glycosides) and anti-oxidants like β-carotene, ascorbic acid, terpenoids, and alkaloids [10]. According to these properties, increased TAC level and decreased MDA level [11] in rabbit seminal plasma were observed due to the effect of GIN as natural antioxidant. Generally, GIN increased the testicular antioxidant enzymes activities (SOD, catalase, and GPx) and has protective effects against the oxidative stress and testicular damage [25]. The present results are in accordance with El-Speiy et al [10], who reported significant decreased in reaction time from 15.9 to 6.9 s in rabbit bucks, fed diet supplemented with GIN powder. Orally administration of rabbit bucks with GIN aqueous extract increased testosterone level from 3.45 to 6.29 ng/mL and increased sperm production in terms of increased concentration, motility and normality of spermatozoa [11].

The mechanisms through which GIN enhances testosterone production as an androgenic agent, are mainly by improving the activities of 3β-hydroxysteroid dehydrogenase, 17α-hydroxylase and 17, 20- lyase, and 17β-hydroxysteroid dehydrogenase in the testis [26]. Also, GIN increase LH release via increasing the level of cholesterol and reducing lipid peroxidation in the testis. Furthermore, GIN normalizes the blood glucose, enhances nitric oxide production, and increases the blood flow into Leydig cells, besides increasing the testicular weight, and recycling testosterone receptors [25]. This action leads to an increasing testosterone profile, which has important role in maintenance of libido and improvement of semen production in rabbit bucks.

It is worthy noting that improvement in sexual desire and semen quality of rabbit bucks treated with BET was associated with increasing the physiological response by reducing body temperature degrees (RT, ST, and ET) which suggest the action of BET in heat regulation. In this respect, BET has been found to reduce body temperatures estimates (RT, ST, and ET) in growing rabbits [27] under a severe heat load. BET may maintain the thermo-neutral state of animals by reversing the heat-induced inhibition of the osmotic equilibrium and maintaining the tertiary structures of macromolecules in the kidney [28].

In parallel with the effect of BET and EVOO on semen production in our study, BET is reported to have antioxidant activity, which may protect different stages of spermatocytes from apoptosis, leading to an increase in sperm quality in rooster [4]. It is indicated that BET plus Vitamin C and E improved oxidative capacity (TAC and MDA) in rooster chickens reared under HS conditions [4]. In addition, olive oil contains omega-3 polyunsaturated fatty acids [12] and has beneficial bioactive components (flavonoids and polyphenols) as bioactive organic molecules. These compounds have antimicrobial, antioxidant and anti-inflammatory properties [13]. The antioxidant status in the testes of rat were improved in term of increased TAC and decreased MDA level by olive leaves extract [12]. These properties of BET and EVOO may explain the improvement occurred in semen quality of rabbit bucks in our study under HS conditions. High motility, normality and concentration of spermatozoa have been associated with the improving fertility rate [29]. Also, total litter size was influenced significantly by number of motile sperm output. Most of sperm parameters are essential for sperm fertilizability in mammals. Therefore, the foregoing results of rabbit does mated by bucks treated with EVOO, BET, and GIN, concerning the improved semen quality and antioxidant status, reflect improving litter size (total and live) as compared to control bucks. These results are proved by El-Speiy et al [10], when bucks were supplemented with GIN. The positive effect of GIN as an enhancer of reproductive capacity of rabbit bucks may be due to its ability to protect mammal cells from oxidation as showed by Ulkowski et al [30].

## CONCLUSION

In spite of dietary supplementation of EVOO, BET, or GIN can improve oxidative stress, sexual desire and semen quality of rabbit bucks, it is found that the GIN supplementation provides the best impact. Based on the obtained results, GIN may assist rabbit bucks to counter negative impacts of oxidative stress, given the increase of motility, vitality, normality, acrosomal and membrane integrities, functional sperm output and fertility as well as improving biochemical constitutes and antioxidant status. Therefore, dietary supplementation with GIN at a level of 200 mg/kg is recommended as a valuable strategy for enhancing the reproductive efficiency of breeding rabbit bucks used in natural mating or artificial insemination.

## Figures and Tables

**Table 1:** Ingredients and chemical analysis of the diet used for feeding rabbit bucks in different experimental treatments

Items	
Ingredient (g/kg)	
Clover hay	400
Barley grain	125
Wheat brain	145
Soybean meal (44% crude protein)	180
yellow corn	100
Molasses	30
Di-calcium phosphate	8
Limestone	5
DL-methionine	1
Sodium chloride	3
Vitamins+minerals^1)^	3
Chemical analysis (%)	
Crude protein	17.20
Crude fiber	13.80
Ether extract	2.70
Nitrogen free extract	57.10
Ash	9.20
Digestible energy (kcal/kg)	2550
Calcium	1.23
Total phosphorus	0.80
Lysine	0.95
Methionine	0.46
Sodium	0.16

1) The vitamin and mineral premix/kg contained Vitamin A, 6,000 IU; Vitamin D_3_, 900 IU; Vitamin E, 40 mg; Vitamin K_3_, 2 mg; Vitamin B_1_, 2 mg; Vitamin B_2_, 4 mg; Vitamin B_6_, 2 mg; Pantothenic acid, 10 mg; Vitamin B_12_, 0.01 mg; Niacin, 50 mg; Folic acid, 3 mg; Biotin, 0.05 mg; Choline, 250 mg; Fe, 50 mg; Mn, 85 mg; Cu, 5 mg; Co, 0.1 mg; Se, 0,1 mg; I, 0.2 mg; and Zn, 50 mg.

**Table 2:** Ambient temperature, relative humidity, and the calculated temperature humidity index during the experimental treatments

Treatment period	AT (°C)	RH (%)	THI
1st June to 30th June	30.93	72.46	29.53
1st July to 31st July	32.80	75.53	31.40
1st August to 30th August	32.66	73.06	31.14
1st September to 30th September	29.53	74.20	28.32

AT, ambient temperature; RH, relative humidity; THI, temperature humidity index.

**Table 3:** Effect of extra virgin olive oil, betaine, and ginger supplementations on body weight, feed intake and body temperature degrees of APRI rabbit bucks during the experimental treatment (n = 10 in each group)

Parameters	Control	Natural antioxidant treatments			p-value^*^		
	
		EVOO	BET	GIN	Treatments	Normality	Control vs Treatments
Bucks performance							
Final BW (kg)	3.20±0.036	3.18±0.046	3.14±0.029	3.18±0.029	0.668	0.191	0.390
Feed intake (g, buck/d)	90.70±1.983	94.20±1.123	91.90±1.286	91.60±1.790	0.459	0.216	0.314
Body temperature degrees (°C)							
Rectal temperature	39.05±0.083^a^	38.95±0.078^a^	38.79±0.053^b^	38.91±0.122^ab^	0.041	0.081	0.028
Skin temperature	38.80±0.066^a^	38.72±0.062^ab^	38.55±0.064^b^	38.76±0.032^ab^	0.018	0.075	0.012
Ear temperature	38.61±0.057^a^	38.41±0.053^ab^	38.30±0.111^b^	38.56±0.021^ab^	0.025	0.070	0.008

APRI, Animal Production Research Institute; EVOO, extra virgin olive oil; BET, betaine; GIN, ginger; BW, body weight.

* p-value of treatments, normality and control vs treatments columns were calculated by the one-way analysis of variance, Shapiro-Wilk test and orthogonal contrasts analysis of variance, respectively.

a,b Means with different superscripts in the same row are significantly different (* p<0.05, Tukey HSD test).

**Table 4:** Effect of extra virgin olive oil, betaine, and ginger supplementations on semen production parameters of APRI rabbit bucks (n = 10 in each group)

Parameters	Control	Natural antioxidant treatments			p-value^*^		
	
		EVOO	BET	GIN	Treatments	Normality	Control vs Treatments
Net semen volume (mL)	0.77±0.016^c^	0.83±0.009^b^	0.85±0.011^ab^	0.89±0.006^a^	0.001	0.155	0.001
Semen pH value	7.11±0.025^b^	7.18±0.025^ab^	7.19±0.015^a^	7.23±0.016^a^	0.002	0.089	0.001
Sperm progressive motility (%)	61.20±1.451^b^	73.50±1.500^a^	73.70±1.422^a^	75.50±1.572^a^	0.001	0.281	0.001
Sperm vitality (%)	65.50±1.013^c^	75.80±1.297^b^	77.40±0.991^ab^	80.30±1.065^a^	0.001	0.160	0.001
Sperm normality (%)	74.90±0.948^c^	80.40±0.871^b^	82.60±0.871^b^	86.90±0.690^a^	0.001	0.576	0.001
Sperm abnormality (%)	25.10±0.948^a^	19.60±0.871^b^	17.40±0.871^b^	13.10±0.690^c^	0.001	0.576	0.001
Sperm cells with abnormal tail (%)	21.00±1.021^a^	16.40±1.097^b^	14.30±0.955^bc^	10.70±0.760^c^	0.001	0.251	0.001
Sperm cells with abnormal head (%)	4.10±0.433^a^	3.20±0.442^ab^	3.10±0.433^ab^	2.40±0.426^b^	0.043	0.173	0.022
Sperm cell concentration (×10^6^/mL)	280.70±5.660^c^	319.80±3.915^b^	334.30±4.786^ab^	341.00±1.972^a^	0.001	0.158	0.001
Intact acrosome (%)	79.60±0.748^b^	83.70±0.715^a^	84.20±0.711^a^	85.50±1.013^a^	0.001	0.276	0.001
Membrane integrity (%)	29.70±0.746^c^	36.50±0.885^b^	42.90±0.737^a^	43.40±0.991^a^	0.001	0.109	0.001
Sperm output (×10^6^/ejaculate)							
Total	215.95±5.574^d^	266.17±5.033^c^	284.86±3.454^b^	303.86±3.137^a^	0.001	0.262	0.001
Motile	132.08±4.318^c^	195.65±5.627^b^	209.95±4.870^ab^	229.48±5.671^a^	0.001	0.392	0.001
Normal	161.88±5.188^d^	214.01±4.768^c^	235.23±3.341^b^	264.07±3.617^a^	0.001	0.273	0.001
Vital	141.41±4.168^c^	202.27±7.027^b^	220.31±2.560^b^	244.06±4.452^a^	0.001	0.384	0.001
Functional	64.656±2.069^c^	119.68±5.518^b^	134.33±4.221^b^	160.12±4.589^a^	0.001	0.173	0.001

APRI, Animal Production Research Institute; EVOO, extra virgin olive oil; BET, betaine; GIN, ginger.

* p-value of treatments, normality and control vs treatments columns were calculated by the one-way analysis of variance, Shapiro-Wilk test and orthogonal contrasts analysis of variance, respectively.

a–d Means with different superscripts in the same row are significantly different (* p<0.05, Tukey HSD test).

**Table 5:** Effect of extra virgin olive oil, betaine, and ginger supplementations on biochemical constitutes, enzyme activity and oxidative capacity in seminal plasma of APRI rabbit bucks (n = 5 in each group)

Parameters	Control	Natural antioxidant treatments			p-value*		
	
		EVOO	BET	GIN	Treatments	Normality	Control vs Treatments
Biochemical constitutes							
Total protein (g/dL)	4.20±0.053^b^	4.58±0.026^a^	4.61±0.020^a^	4.62±0.021^a^	0.001	0.098	0.0001
Albumin (AL, g/dL)	1.97±0.025^b^	2.04±0.030^ab^	2.06±0.022^ab^	2.13±0.026^a^	0.008	0.840	0.004
Globulin (GL, g/dL)	2.22±0.061^b^	2.49±0.039^a^	2.53±0.034^a^	2.55±0.019^a^	0.001	0.083	0.0001
AL/GL ratio	0.89±0.032	0.81±0.020	0.81±0.013	0.85±0.023	0.042	0.166	0.0220
Total lipids (mg/dL)	410.40±3.828^c^	436.40±2.315^b^	439.80±1.593^b^	450.20±1.655^a^	0.001	0.094	0.0001
Initial semen fructose	183.80±2.154^b^	222.60±2.181^a^	226.80±2.289^a^	227.20±1.773^a^	0.001	0.090	0.001
Enzyme activity (IU/L)							
AST	30.80±1.240^a^	23.20±1.984^b^	21.80±1.280^b^	18.40±1.208^b^	0.002	0.407	0.0001
ALT	23.40±1.208^a^	17.40±1.077^b^	15.60±1.029^cb^	13.60±1.326^c^	0.001	0.859	0.0001
ALP	56.60±1.435^b^	66.20±1.462^a^	67.00±1.140^a^	67.80±1.240^a^	0.001	0.103	0.0001
ACP	30.60±1.964	32.60±1.503	35.40±1.964	35.80±1.319	0.146	0.375	0.060
Oxidative capacity							
TAC (mmol/L)	0.84±0.018^b^	0.93±0.019^b^	1.10±0.035^a^	1.13±0.020^a^	0.001	0.213	0.001
GSH (mg/dL)	10.40±1.208^b^	15.60±1.208^a^	16.60±1.077^a^	17.40±1.435^a^	0.004	0.956	0.001
GPX (mg/dL)	4.20±0.800	4.53±0.177	4.88±0.086	4.90±0.035	0.596	0.064	0.248
SOD (IU)	5.68±0.166	5.85±0.050	5.94±0.026	5.96±0.041	0.143	0.087	0.084
GST (IU)	1.08±0.025^b^	1.25±0.016^a^	1.31±0.031^a^	1.32±0.024^a^	0.001	0.210	0.001
MDA (nmol/mL)	22.20±1.496^a^	17.20±1.157^b^	13.80±1.280^b^	13.60±2.227^b^	0.004	0.962	0.001

APRI, Animal Production Research Institute; EVOO, extra virgin olive oil; BET, betaine; GIN, ginger; AST, aspatate amino transferase; ALT, alanine amino transferase; ALP, alkaline phosphatase; ACP, acid phosphatase; TAC, total antioxidant capacity; GSH, glutathione content; GPx, glutathione peroxidase; SOD, superoxide dismutase; GST, glutathione S-transferase; MDA, malondialdehyde.

* p-value of treatments, normality and control vs treatments columns were calculated by the one-way ANOVA, Shapiro-Wilk test and orthogonal contrasts ANONA, respectively.

a–c Means with different superscripts in the same row are significantly different (* p<0.05, Tukey HSD test).

**Table 6:** Odds ratio estimates for pregnancy and parturition cases in extra virgin olive oil, betaine, and ginger groups compared to the control

Parameters	Odds ratio	(95% CI)	p-value
Pregnancy rate			
EVOO	1.308	(0.571 – 2.671)	0.605
BET	4.000	(1.541 – 5.691)	0.017
GIN	5.000	(1.661 – 7.331)	0.008
Parturition rate			
EVOO	1.625	(0.569 – 3.714)	0.539
BET	3.500	(1.192 – 5.443)	0.129
GIN	5.749	(1.051–9.590)	0.056

EVOO, extra virgin olive oil; BET, betaine; GIN, ginger.

**Table 7:** Reproductive performance of APRI rabbit does naturally mated by rabbit bucks received extra virgin olive oil, betaine, and ginger in the experimental treatments

Parameters	Control	Natural antioxidant treatments			p-value^*^		
	
		EVOO	BET	GIN	Treatments	Normality	Control vs Treatments
Total mated rabbit does (n)	30	30	30	30	-	-	-
Pregnant rabbit does (n)	15	17	24	25	-	-	-
Delivered rabbit does (n)	10	13	21	23	-	-	-
Total litter size at birth (n)	4.40±0.426^b^	5.92±0.309^a^	6.23±0.283^a^	6.30±0.352^a^	0.005	0.110	0.001
Live litter size at birth (n)	3.10±0.314^c^	4.38±0.180^b^	5.23±0.315^ab^	5.82±0.336^a^	0.001	0.199	0.0001
Litter size at weaning (n)	2.30±0.260^c^	3.46±0.243^b^	4.38±0.304^a^	4.91±0.273^a^	0.001	0.228	0.0001
Viability rate at birth (%)	72.80±6.695^b^	75.76±4.034^b^	83.47±2.775^ab^	92.67±2.269^a^	0.001	0.098	0.024
Viability rate at weaning (%)	75.50±6.106	80.38±5.813	84.19±3.348	85.45±2.801	0.407	0.111	0.174

APRI, Animal Production Research Institute; EVOO, extra virgin olive oil; BET, betaine; GIN, ginger.

* p-value of treatments, normality and control vs. treatments columns were calculated by the one-way ANOVA, Shapiro-Wilk test and orthogonal contrasts ANONA, respectively.

a–c Means with different superscripts in the same row are significantly different (* p<0.05, Tukey HSD test).
